# Reply to: Immortal time bias in the analysis of drug prescription trajectories

**DOI:** 10.1038/s41746-022-00724-4

**Published:** 2022-12-23

**Authors:** Laust Hvas Mortensen, Søren Brunak

**Affiliations:** 1 Statistics Denmark, DK-2100 Copenhagen, Denmark; 2 Section of Social Medicine, Department of Public Health, University of Copenhagen, DK-1014 Copenhagen, Denmark; 3 Novo Nordisk Foundation Center for Protein Research, Faculty of Health and Medical Sciences, University of Copenhagen, DK-2200 Copenhagen, Denmark

**Keywords:** Health care, Computational biology and bioinformatics

**replying to** Daniel Mølager Christensen, Gunnar Gislason, Thomas Gerds *npj Digital Medicine* 10.1038/s41746-022-00722-6 (2022)

We thank Christensen et al. for their perspective on our recent paper, including the specific comments regarding the inappropriate accounting of time at risk in a specific set of survival analyses included in the paper. We welcome the critique and acknowledge that being alive is a prerequisite for changing treatment, thus biasing the survival analysis by including immortal time. We should have taken account of this in the analyses of this particular finding in the paper.

In our view, the primary contribution of our paper is to describe how patients follow trajectories. We agree with Christensen et al. that the findings in our paper are descriptive and cannot readily be interpreted as causal effects, but we trust that this is clear to the reader. We believe that descriptive analyses are of importance, particularly in situations where good identification strategies for causal effects are difficult to arrive at. Consequently, it is more than likely that there are systematic unobserved differences between individuals that follow different trajectories. For example, it seems very likely that progression from a first line treatment to a second line treatment will be prompted by change in treatment response. This treatment response is not directly observed. The treatment response itself is likely influenced by the nature and severity of the disease(s) that the patient had when the drug was prescribed. This information is also not modelled. We expect future work to extend the models that we have presented to incorporate more information of patient characteristics to reduce confounding and increase the comparability of patients.

